# Genetic Divergence and Relationship Among *Opisthopappus* Species Identified by Development of EST-SSR Markers

**DOI:** 10.3389/fgene.2020.00177

**Published:** 2020-02-28

**Authors:** Min Chai, Hang Ye, Zhi Wang, Yuancheng Zhou, Jiahui Wu, Yue Gao, Wei Han, En Zang, Hao Zhang, Wenming Ru, Genlou Sun, Yling Wang

**Affiliations:** ^1^School of Life Sciences, Shanxi Normal University, Linfen, China; ^2^Triticeae Research Institute, Shanxi Academy of Agricultural Science, Linfen, China; ^3^Changzhi University, Changzhi, China; ^4^Department of Biology, Saint Mary’s University, Halifax, NS, Canada

**Keywords:** *Opisthopappus*, transcriptome sequencing, EST-SSRs development, population genetics, phylogenetic relationship

## Abstract

*Opisthopappus* Shih is an endemic and endangered genus restricted to the Taihang Mountains that has important ornamental and economic value. According to the Flora Reipublicae Popularis Sinicae (FRPS, Chinese version), this genus contains two species (*Opisthopappus longilobus* and *Opisthopappus taihangensis*), whereas in the *Flora of China* (English version) only one species *O. taihangensis* is present. The interspecific phylogenetic relationship remains unclear and undefined, which might primarily be due to the lack of specific molecular markers for phylogenetic analysis. For this study, 2644 expressed sequence tag-simple sequence repeats (EST-SSRs) from 33,974 unigenes using a *de novo* transcript assembly of *Opisthopappus* were identified with a distribution frequency of 7.78% total unigenes. Thereinto, mononucleotides (1200, 45.39%) were the dominant repeat motif, followed by trinucleotides (992, 37.52%), and dinucleotides (410, 15.51%). The most dominant trinucleotide repeat motif was ACC/GGT (207, 20.87%). Based on the identified EST-SSRs, 245 among 1444 designed EST-SSR primers were selected for the development of potential molecular markers. Among these markers, 63 pairs of primers (25.71%) generated clear and reproducible bands with expected sizes. Eventually, 11 primer pairs successfully amplified all individuals from the studied populations. Through the EST-SSR markers, a high level of genetic diversity was detected between *Opisthopappus* populations. A significant genetic differentiation between the *O. longilobus* and *O. taihangensis* populations was found. All studied populations were divided into two clusters by UPGMA, NJ, STRUCTURE, and PCoA. These results fully supported the view of the FRPS, namely, that *O. longilobus* and *O. taihangensis* should be regarded as two distinct species. Our study demonstrated that transcriptome sequences, as a valuable tool for the quick and cost-effective development of molecular markers, was helpful toward obtaining comprehensive EST-SSR markers that could contribute to an in-depth assessment of the genetic and phylogenetic relationships between *Opisthopappus* species.

## Introduction

*Opisthopappus* Shih is a perennial herbaceous genus of the Asteraceae family, which is endemic to and endangered within China, that grows primarily in the cliff cracks and rock gaps of the Taihang Mountains (Shih, 1979; Ding and Wang, 1997; Wu et al., 1999; Hu et al., 2008). This genus exhibits excellent drought tolerance and leanness resistance that make it an important wild *Chrysanthemum* resource (Chai et al., 2018). Being a diploid, *Opisthopappus* is a 2A asymmetrical karyotype that has an intimate relationship with the Artemiisinae subtribe (Zhao et al., 2009). AFLP analysis has revealed that *Opisthopappus* is clustered with a majority of *Ajania* taxa, located on the basal branch of *Dendranthema* and *Ajania*. This indicates that *Opisthopappus* might have been a more primitive group during the eastward spread and evolutionary process of *Dendranthema* ancestor groups (Zhao et al., 2010).

According to the Flora Reipublicae Popularis Sinicae (FRPS)^1^ (Shih and Fu, 1983), *Opisthopappus* contains two species, *Opisthopappus longilobus* Shih and *Opisthopappus taihangensis* (Ling) Shih. The morphological differentiation between these two species presents mainly on the leaves and bracts. For *O. longilobus*, which has hairless leaves, there is one pinnatisect, except the basal stem leaves, and a pair of bracteal leaves beneath the involucres. For *O. taihangensis*, there is appressed puberulent on both surfaces of the leaves, two pinnatisect stem leaves, and no bracteal leaves. Nevertheless, according to the *Flora of China*^2^ (Wu et al., 2013), *O. taihangensis* was the only one species in the genus of *Opisthopappus* and *O. longilobus* was merged into it. Its basal leaves are one- or two-pinnatisect, both surfaces sparsely pubescent or glabrous, and the stem leaves are similar to the basal leaves, uppermost leaves pinnatifid.

Based on the observation on the pollen morphology of *Opisthopappus*, the results showed the pollen of *O. longilobus* at spine length and density, ostiole, density, colpus depth is more highly evolved than that of *O. taihangensis* (Gao et al., 2011). The micromorphological characteristics of leaves revealed that these two species differed in their characteristics and density of glandular hairs, size and density of stomata, anticlinal wall patterns, wax characteristics, as well as the size and density of salt vesicles (Jia and Wang, 2015). A comparison between the biological characteristics of *O. longilobus* and *O. taihangensis* in their natural habitats and artificial populations indicated stable inheritable characters and significant differences in their leaf morphologies and other features (Hu et al., 2008). And above all, the evolutionary relationships and taxonomic status of the species within *Opisthopappus* remain debated and inconclusive. Further investigations and analysis are required to definitively determine whether *O. longilobus* and *O. taihangensis* should be regarded as two distinct species or a single species.

Our previous research endeavored to explore the relationships between *Opisthopappus* species through various molecular markers, such as Inter-Simple Sequence Repeat (ISSR), Sequence Related Amplified Polymorphism (SRAP), Internal Transcribed Spacer (ITS), and chloroplast gene sequences (Wang, 2013; Wang and Yan, 2013, 2014; Wang et al., 2015). However, cluster analysis revealed that neither *O. longilobus* nor *O. taihangensis* formed a monophyletic clade (Wang, 2013; Wang and Yan, 2013), particularly, some populations of *O. longilobus* always gathered with *O. taihangensis* populations. These cluster analysis results indicated that the two species would seemingly be merged into one species, or the limited gene data offered from the above molecular markers might not be sufficient for phylogeny analysis. Zhang et al. (2019) also pointed that the phylogenetic relationships and genetic components between species were unclear due to the limited detection capacity of specific or unique nuclear gene sequences. Therefore, the development of transferable and orthologous molecular markers between phylogenetically related species of *Opisthopappus* is essential and critical.

Recently, RNA sequencing (RNA-Seq) has been employed in the development of a high-throughput sequencing technology that has the ability to profile the entire transcriptome of any organism, particularly for non-model plants that lack sequenced genomic data (Gross et al., 2013; Wu et al., 2014; Du et al., 2015; Zhang et al., 2019). Meanwhile, transcriptome sequencing holds great potential as a platform for the generation of molecular markers (Martin et al., 2013). Further, *de novo* RNA-Seq assembly can provide a cost-effective approach to identify molecular markers, such as microsatellite markers (SSRs) and single-nucleotide polymorphisms (SNPs) (Davey et al., 2011; Karan et al., 2012; Chen et al., 2013; Zhang et al., 2019). What is more, both of SSRs and SNPs markers are codominant and the reflection of specific segment or sequence information, which are recommended by UPOV-BMT molecular testing guidelines as optimal molecular markers for authenticity identification of varieties and database construction (UPOV-BMT, International Union for the Protection of New Varieties of Plants-Molecular Biology Technology Working Group 2007^3^). Based on the predicted SNPs, we have successfully developed eight SNP markers for population genetic analysis in *Opisthopappus* (Chai et al., 2018). However, with the help of the new technology and effective method, more information and further evidences are needed for more comprehensively and accurately solving the problem on the taxonomy of *Opisthopappus*.

Simple sequence repeats derived from ESTs (EST-SSRs) identified from transcribed RNA sequences typically contain mono-, di-, tri-, tetra-, penta-, or hexa-nucleotide units, and possess abundant information, codominance, and loci specificity (Song et al., 2012; Zhou et al., 2016). With codominant features between distantly related species, EST-SSRs cross-transferability can shed new light on the evolution of plant genomes, changes in gene location, and genome organization (Bazzo et al., 2018; Zhang et al., 2019). Moreover, this transferability provides a cost-effective source of markers for related species, which is important for taxa with low microsatellite frequencies, or for those whose microsatellites are difficult to isolate (Bazzo et al., 2018; Zhang et al., 2019). Previous transcriptome sequences in *Opisthopappus*, which generated and identified 33,974 unigenes and more than 3410 differentially expressed transcripts (DETs), provided crucial genetic resources and sequence data toward the development of EST-SSR markers (Chai et al., 2018).

Therefore, in this study, we developed EST-SSR markers from the *Opisthopappus* transcriptome and characterized their frequency, distribution, and function, which were further employed to elucidate the genetic differentiation and phylogenetic relationships between *O. longilobus* and *O. taihangensis*.

## Materials and Methods

### Development of EST-SSR Markers From *Opisthopappus* Transcriptome

The cDNA libraries were constructed using *Opisthopappus* tissue samples, and sequenced based on next-generation sequencing (NGS) (Chai et al., 2018). The obtained raw reads were preprocessed to filter adaptor sequences, low-quality sequences, and reads with qualities of less than Q30 using the FASTX toolkit (Gordon and Hannon, 2010). Following the removal of raw reads, high-quality clean reads were assembled *de novo* into contigs and unigenes using a short read assembling program called Trinity RNA-Seq Assembler (Haas et al., 2013). The assembled unigene sequences were directly identified by using MicroSatellite (MISA) identification tools (Thiel et al., 2003), for which the parameters were set at mono-, di-, tri-, tetra-, penta-, and hexa-nucleotide motifs as a minimum repeat number of 12, 6, 5, 5, 4 and 4, respectively. Based on the MISA results, the EST-SSR primers were designed using Primer3 software (Untergasser et al., 2012).

### Plant Materials for Genetic Analysis

**FIGURE 1:**
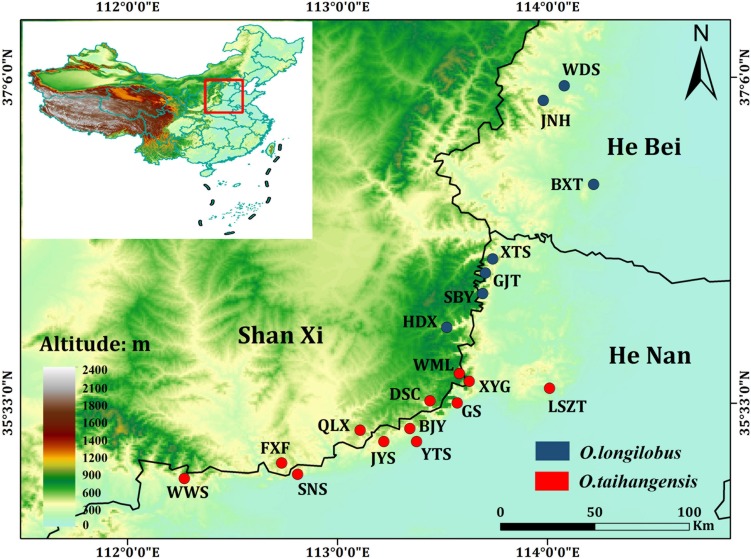
Geographic distribution of the 19 sampled populations of *Opisthopappus* in China. For population abbreviations, see Table 1 for details.

Nineteen populations of *Opisthopappus* were sampled, including 12 populations of *O. taihangensis* and 7 populations of *O. longilobus*, which were collected during August and September of 2017 that essentially covered their distribution ranges (Table 1 and Figure 1). Each individual from the same population was collected from different locations at least 5 m apart. Fresh young leaves were selected in each of the sample sites, where 10–15 individuals from each population were collected, for a total of 214 samples from 19 populations. The collected samples were quickly dried with silica gel and stored at 20°C for future use. A global positioning system (GPS) was employed to demarcate each sample site and record the longitude, latitude, and altitude of each population.

**TABLE 1:** Sampling location information and genetic diversity of *Opisthopappus* based on EST-SSR markers.

**Population**	**Species**	**Location**	**No.**	**E (°)**	**N (°)**	**Alt**	**Na**	**Ne**	***H***	***I***	**PPB (%)**
HDX	*O. longilobus*	Hongdouxia, Shanxi	11	113.52	35.91	1070	1.282	1.232	0.2326	0.248	63.35
XTS	*O. longilobus*	Xiantaishan, Henan	10	113.73	36.18	873	0.774	1.131	0.0438	0.139	34.71
WDS	*O. longilobus*	Wudangshan, Hebei	12	114.08	37.06	1160	0.854	1.234	0.1311	0.201	36.89
JNH	*O. longilobus*	Jingnianghu, Hebei	12	113.98	36.99	650	0.981	1.218	0.0645	0.201	46.84
BXT	*O. longilobus*	Beixiangtang, Hebei	15	114.22	36.59	520	0.350	1.045	0.0315	0.056	17.23
SBY	*O. longilobus*	Shibanyan, Henan	15	113.68	36.07	720	1.226	1.144	0.1049	0.189	61.17
GJT	*O. longilobus*	Gaojiatai, Henan	12	113.69	36.11	805	1.138	1.160	0.112	0.196	56.80
Species level							1.937	1.356	0.137	0.327	96.84
SNS	*O. taihangensis*	Shennongshan, Henan	14	112.81	35.21	1028	1.449	1.377	0.1466	0.350	71.60
FXF	*O. taihangensis*	Fuxifeng, Shanxi	14	112.73	35.26	1120	1.284	1.295	0.0806	0.282	63.35
GS	*O. taihangensis*	Guanshan, Henan	14	113.57	35.55	609	1.539	1.352	0.1691	0.333	75.97
WWS	*O. taihangensis*	Wangwushan, Henan	12	112.27	35.19	1521	1.214	1.306	0.1817	0.287	59.22
WML	*O. taihangensis*	Wangmangling, Shanxi	12	113.58	35.69	1421	1.092	1.237	0.1454	0.235	53.64
LSZT	*O. taihangensis*	Linshizuting, Henan	13	114.01	35.62	530	0.976	1.252	0.1463	0.229	46.36
BJY	*O. taihangensis*	Baijiayan, Henan	15	113.42	35.42	866	1.068	1.234	0.1448	0.234	52.18
YTS	*O. taihangensis*	Yuntaishan, Henan	15	113.43	35.42	1011	1.374	1.304	0.1845	0.298	67.96
XYG	*O. taihangensis*	Xiyagou, Shanxi	12	113.59	35.64	1268	1.090	1.198	0.1269	0.211	53.16
QLX	*O. taihangensis*	Qinglongxia, Shanxi	13	113.17	35.42	841	1.274	1.344	0.1148	0.309	60.68
JYS	*O. taihangensis*	Jingyingsi, Henan	10	113.22	35.39	1006	1.444	1.373	0.1292	0.350	71.36
DSC	*O. taihangensis*	Dashuangcun, Shanxi	11	113.44	35.56	868	1.214	1.323	0.0706	0.295	57.77
Species level							1.867	1.223	0.103	0.271	93.20
*Opisthopappus*							2.000	1.300	0.2028	0.344	100.00

*No., sample number; E, longitude; N, latitude; Alt, altitude; Na, observed number of alleles; Ne, effective number of alleles; *H*, Nei’s genetic diversity; *I*, Shannon’s information index; PPB, the percentage of polymorphic loci/band.*

### DNA Extraction and PCR Amplification

The genomic DNA was extracted from leaves though modified CTAB methods (Doyle et al., 1987). The quantity and quality of the DNA samples were determined using a NanoDrop ND1000 spectrophotometer (Thermo Scientific, Waltham, MA, United States) and 0.8% agarose gel electrophoresis, which were then diluted to 30 ng/μl and stored at −20°C until further use.

Two hundred twenty-five EST-SSR primer pairs were initially designed based on the potential SSR loci identified in transcriptome. Subsequently, 63 primer pairs were selected to amplify 19 random DNA samples (one for each population) for the pre-experiments (primer screening). These abided by the following principles: the primer length was controlled between 18 and 25 bp, the GC content was from 40 to 60%, and the annealing temperature was from 50 to 60°C. Hairpin structures, dimers, hat structures, and mismatches were avoided as much as possible.

The primers were synthesized by Shanghai Sangon Biological Engineering Technology (Shanghai, China). Among these 63 primer pairs, 11 primers were ultimately selected based on high transferability, polymorphism, and repeatability to generate clear and reproducible bands with expected sizes, which were used to further amplify all individuals of the *Opisthopappus* populations (Table 2).

**TABLE 2:** Genetic diversity index of the used EST-SSR markers in *Opisthopappus*.

**Primer**	**SSRs**	**Forward primer**	**Reverse primer**	**Tm (°C)**	***T***	**TP**	**PPB (%)**	**He**	**Ho**	**PIC**
1	ACA(6)	GTTCCACCACCATTG	TGCCTAAGAGAACAGATA	56.15	31	31	49.07	0.147	0.154	0.3667
	CAA(6)	AGTTTGTGAG	ATGAGAT							
2	ACA(6)	GTTCCACCACCATTG	GATGGTCAATCAAAGAGT	56.8	34	34	54.95	0.136	0.143	0.2878
	CAA(6)	AGTTTGTGAG	GTGGCTA							
3	ACA(6)	GTTCCACCACCATTG	AGGAATGGGAATACAAA	56.85	27	27	49.12	0.131	0.137	0.3372
	CAA(6)	AGTTTGTGAG	GACGAAAA							
4	AAAC(5)	CGGAGAAATAGTAAGTCG	GAGTAGGCATGAGTGGGT	49.7	52	52	62.65	0.183	0.192	0.3860
5	AAAC(5)	ACTCCTACAGTGTCGGTG	AATACTACTGCTACTGGCTCTT	53.65	39	39	66.94	0.191	0.200	0.4411
6	AGCA(5)	GAGTGGTGTGGTGTTTTACTGTCGT	ACGATTTGTGCTGTTCTGGGAGTGG	57.35	31	31	46.01	0.116	0.122	0.3411
7	GAA(6)	CAAGGTCGTCAACTCATAA	CTGCTGTGATGGGAAAGA	51.3	44	44	61.96	0.171	0.180	0.3863
8	GAA(6)	ATGTTCCGTTTGACGAGA	GCTACTCCTGACCGACTT	53.85	47	47	48.82	0.138	0.145	0.3756
9	AGCA(5)	AACAATAAGTCCGCCACA	ACTCACCCAAGAATACATCG	53.45	38	38	49.31	0.166	0.174	0.3958
10	AGCA(5)	ATGTGCTTTACCCTTACC	GGAGAAGAGTAAAGAGGAGGCT	53.75	40	40	54.47	0.171	0.180	0.3817
11	AGCA(5)	GATTTGTGCTGTTCTGGGAG	GAGGCTGGGATTTGGGTT	55.7	29	29	58.26	0.136	0.143	0.2919

**T*, total number of amplified bands; TP, total number of polymorphic bands; PPB, percentage of polymorphism; He, expected heterozygosity; Ho, observed heterozygosity; PIC, polymorphic information content.*

Each 20 μL PCR reaction contained 10 μL 2× MasterMix, 0.7 μL 10 μM forward primer, 0.7 μL 10 μM reverse primer, 3 μL DNA template (30 ng/μL), and 5.6 μL H_2_O. The following PCR conditions were employed: 95°C pre-denaturation for 3 min; 35 cycles of 95°C denaturation for 60 s, 50–60°C annealing for 60 s, and 72°C elongation for 5 min; and a final step at 72°C elongation for 10 min, then held at 4°C. The PCR products were detected using 12% polyacrylamide gel electrophoresis with 400 V for 3–4 h, which were then stained and photographed.

### Data Analysis

The presence or absence of each SSR fragment was coded as “1” and “0,” respectively, and only clear and reproducible bands were considered. For the neutrality test of microsatellite alleles, we used the Bayesian-based outlier analysis of the multinomial-Dirichlet model (BayeScan analysis) to assess the *F*_*ST*_ distribution among all alleles by BAYESCAN v 2.1 (Beaumont and Nichols, 1996; Hsiung et al., 2017).

Genetic diversity parameters, including the observed number of alleles (Na), effective number of alleles (Ne), Nei’s genetic diversity (*H*), Shannon’s information index (*I*), and the percentage of polymorphic bands (% PPB) were calculated using the POPGENE v 1.31 program (Yeh et al., 1999).

A principal coordinate analysis (PCoA) was constructed using GENALEX v 6.5 (Peakall and Smouse, 2012) and visualized with the R package “ggplot2” (Wickham, 2015). The unweighted pair-group method with arithmetic means (UPGMA) tree and neighbor-joining (NJ) trees were constructed using MEGA v 7 (Kumar et al., 2016), through which the probabilities for each node was assessed by PUAP with 1000 replicates bootstrap (Swofford, 2000). The analysis of molecular variance (AMOVA) was employed to investigate the total genetic variation between and within species, and among and within populations using GENALEX v 6.5 (Peakall and Smouse, 2012).

Bayesian clustering analysis (BCA) was utilized to examine the similarity and divergence of genetic components between populations, and was performed using STRUCTURE 2.3.4 (Hubisz et al., 2009). The analysis was run for *K*-values ranging from 1 to 19 possible clusters with 15 independent runs each and a burn-in of 10,00,000 iterations and 20,00,000 subsequent MCMC steps to evaluate consistency. The combination of an admixture and a correlated-allele frequencies model was used. The posterior probability number of grouping was evaluated using △*K* by STRUCTURE HARVESTER 0.6.94 (Earl, 2012). These runs were aligned and summarized using CLUMPP 1.1.2 (Jakobsson and Rosenberg, 2007), and the visualization of the results was plotted using DISTRUCT 1.1 (Rosenberg, 2004).

Pearson correlation analysis was used to test the correlations between the genetic parameters and geographic factors (longitude, latitude, altitude). Correlations between pairwise genetic distance and adjusted pairwise geographic distances were calculated with GENALEX v 6.5 (Peakall and Smouse, 2012) using the mantel test with 9999 permutations. The generalize linear regression model (GLRM) was conducted between the two distances (Nelder and Wedderburn, 1972).

## Results

### Characterization and Distribution of EST-SSR Markers

Through the removal of adaptors, approximately 34.9 million clean reads were obtained. The average Q30 was >95%, which reflected the high quality of the sequencing data, whereas the percentage of unknown nucleotides was only ∼0.01%. The obtained unigenes were 33,974 having paired-end reads with average lengths of 801 bp. Among these unigenes, the lengths of 14,810 unigenes (43.59%) ranged from 200 to 500 bp, and 1893 (5.57%) unigenes were longer than 2 kb. Of the 33,974 unigenes, all were successfully annotated in the NR and GO databases. About 990 of the unigenes (2.91%) were simultaneously annotated in all of the databases (NR, NCBI non-redundant protein sequences; Go, Gene Ontology; KEEG, Kyoto Encyclopedia of Genes and Genome; eggNOG, evolutionary genealogy of genes: non-supervised Orthologous Groups; Swiss-Prot) (Supplementary Table S1).

A total of 2644 putative EST-SSRs were identified from 33,974 unigenes (Supplementary Table S2). Among these potential SSRs, five types of motifs were identified: 1200 (45.39%) mono-nucleotide, 410 (15.51%) di-nucleotide, 992 (37.52%) tri-nucleotide, 39 (1.48%) tetra-nucleotide, and 3 (0.11%) penta-nucleotide (Supplementary Table S2).

The repeat numbers for di-nucleotides, tri-nucleotides, tetra-nucleotides varied from 6 to 11, from 5 to 11, and from 6 to 8, respectively. The repeat number for penta-nucleotides was only 6 (Table 3 and Supplementary Table S2). On average, the unigenes contained one SSR per 10.30 kb and a total of 620 (23.45%) SSRs were repeated more than five times, whereas 277 (10.48%) SSRs were repeated more than 15 times. Among the SSRs identified, the AC/GT (268, 10.14%), AG/CT (94, 3.56%), AT/AT (44, 1.67%), ACC/GGT (207, 7.83%), ATC/ATG (185, 7.00%), AAG/CTT (121, 4.58%), AAC/GTT (111, 4.20%), and AGC/CTG (102, 3.86%) motifs were more prevalent than the other motifs.

**TABLE 3:** Summary of the different repeat units of identified EST-SSRs.

**Number of repeat unit**	**Mono-**	**Di-**	**Tri-**	**Tetra-**	**Penta-**	**Total**	**Percentage (%)**
5	0	0	620	0	0	620	23.45
6	0	201	250	32	3	486	18.38
7	0	86	106	6	0	198	7.49
8	0	47	14	0	0	61	2.31
9	0	30	0	1	0	31	1.17
10	415	32	1	0	0	448	16.94
11	250	14	1	0	0	265	10.02
12	112	0	0	0	0	112	4.24
13	76	0	0	0	0	76	2.87
14	70	0	0	0	0	70	2.65
≥15	277	0	0	0	0	277	10.48
Total	1200	410	992	39	3	2644	100

### Genetic Analysis of *Opisthopappus* Species

There were total 420 alleles generated by 11 EST-SSR primers across 214 samples. Eight alleles were removed with the neutrality tests by BAYESCAN 2 and the remaining 412 alleles used for further genetic population assessment and divergence tests (Supplementary Figure S1).

The percentage of polymorphisms (PPB), expected heterozygosity (He), and observed heterozygosity (Ho) ranged from 46.01 to 66.94, 0.116 to 0.191, and 0.122 to 0.200%, respectively. The pair primer 5 performed the highest PPB (66.94%) and PIC (0.4411) value (Table 2).

For the genus *Opisthopappus*, the PPB was 100%. Among all populations, the observed allele number (Na) ranged from 0.35 to 1.539, with an average of 1.13805. The expected allele number (Ne) ranged from 1.045 to 1.377, with an average of 1.2505. Nei’s genetic diversity (*H*) ranged from 0.0315 to 0.2326, with an average of 0.128185. Shannon diversity index (*I*) was from 0.056 to 0.35, with an average of 0.24435.

In general, *O. longilobus* had a relatively higher level of genetic diversity (Na = 1.937, Ne = 1.356, *H* = 0.137, *I* = 0.327, PPB = 96.84%) than *O. taihangensis* (Na = 1.867, Ne = 1.223, *H* = 0.103, *I* = 0.271, PPB = 93.20%). By comparing means, the results showed that the average value of diversity parameters was significantly different between *O. longilobus* and *O. taihangensis*, except for the Nei’s genetic diversity index (*H*) (Table 1 and Supplementary Table S3).

Analysis of molecular variance (Table 4) was employed to evaluate variance components between and within different populations and species. The results revealed that 67% of the total variation occurred within *Opisthopappus* populations, whereas 33% of the total variation existed between the genus populations. For each species, variations also significantly occurred within populations of both *O. longilobus* (62%) and *O. taihangensis* (74%). This was consistent with the hierarchical AMOVA results, which revealed that most of the genetic variation resided within populations (65%) rather than among groups (7%).

**TABLE 4:** Analysis of molecular variance (AMOVA) based on pairwise differences for *Opisthopappus*.

	**Source**	**df**	**SS**	**MS**	**Estimated variance**	**Variance percentage (%)**	
*O. longilobus*	Among Pops	6	1642.795	273.799	20.035	38	*F*_*ST*_ = 0.380
	Within Pops	78	2548.381	32.672	32.672	62	*P* = 0.001
	Total	84	4191.176		52.707	100	
*O. taihangensis*	Among Pops	11	2614.417	237.674	17.589	26	*F*_*ST*_ = 0.261
	Within Pops	117	5821.738	49.758	49.758	74	*P* = 0.001
	Total	128	8436.155		67.348	100	
*Opisthopappus*	Among Pops	18	5023.073	279.06	21.042	33	*F*_*ST*_ = 0.329
	Within Pops	195	8370.119	42.924	42.924	67	*P* = 0.001
	Total	213	13,393.192		63.966	100	
Hierarchical AMOVA	Among Groups	1	765.86	765.86	4.829	7	*F*_*CT*_ = 0.073
based on two groups	Among Pops	17	4257.213	250.424	18.592	28	*F*_*SC*_ = 0.302
	Within Pops	195	8370.119	42.924	42.924	65	*F*_*ST*_ = 0.353
(*O. longilobus* and *O. taihangensis*)	Total	213	13,393.192		66.345	100	*P* = 0.001

**F*_*CT*_, genetic differentiation among groups; *F*_*SC*_, genetic differentiation among populations within groups; *F*_*ST*_, genetic differentiation among populations.*

**FIGURE 2:**
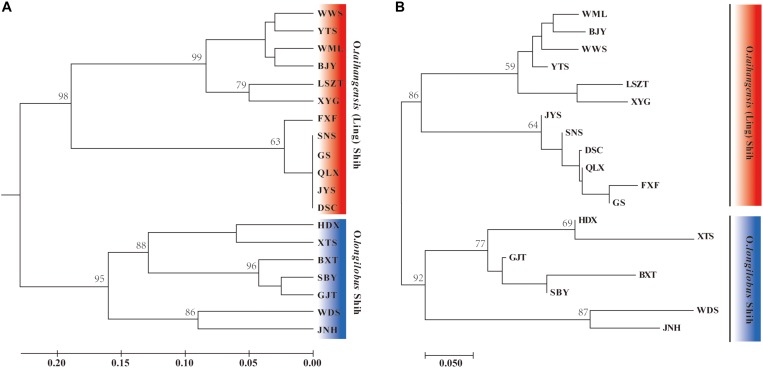
Dendrogram of *Opisthopappus* populations, generated using UPGMA **(A)** and NJ **(B)** cluster analysis. Bootstrap analysis was applied using 1000 replicates and only bootstrap values (%) >50 were given. Nineteen populations were clustered into two groups corresponding to *O. longilobus* and *O. taihangensis* in both UPGMA and NJ trees.

**FIGURE 3:**
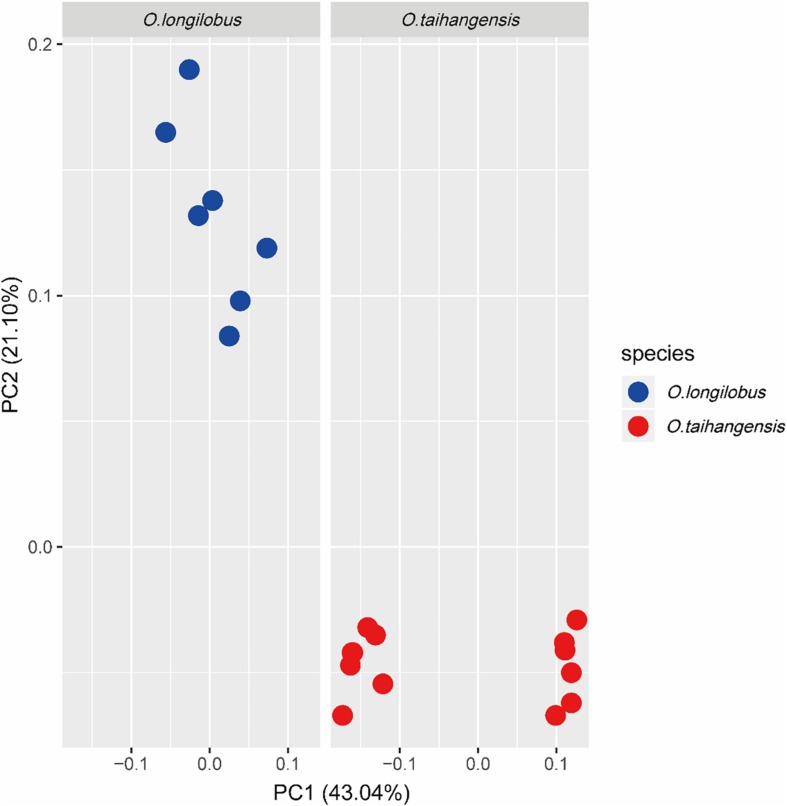
The principal coordinates analysis (PCoA) for *Opisthopappus*. PC1 explained 43.04% variations of genetic components and PC2 explained 21.10% variations of genetic components. The *O. longilobus* and the *O. taihangensis* could be divided into two different groups corresponding to their distribution on axis1 and axis2.

Clustering analysis well distinguished all populations into two major clusters in both UPGAM and NJ tree (Figure 2): *O. taihangensis* cluster and *O. longilobus* cluster. The results we observed from the PCoA were in agreement with the clustering analysis (Figure 3). The first two principal coordinates detected 64.14% of the total variation. The first coordinate axis explained 43.04% of the total genetic components, whereas the second coordinate axis explained 21.10% of the genetic components. A moderate but clear separation between the populations of different species was observed (Figure 3).

**FIGURE 4:**
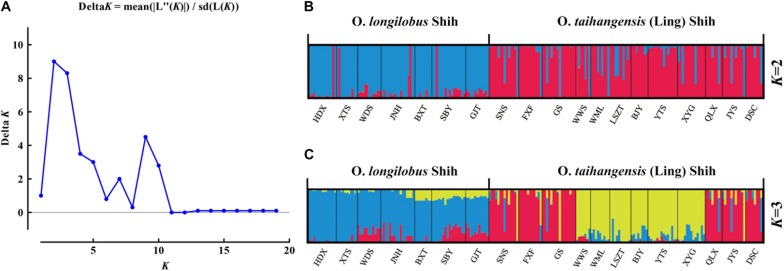
Results of the Bayesian clustering analysis conducted using STRUCTURE. **(A)** The Δ*K* plot shows that *K* = 2 got the highest Δ*K-*value and *K* = 3 got the second-best Δ*K-*value, meaning that the most probable grouping number could be two or three. **(B)** Estimated genetic structure for *K* = 2 obtained for *Opisthopappus*. **(C)** Estimated genetic structure for *K* = 3 for *Opisthopappus*.

The genetic structures of 19 populations of *Opisthopappus* were analyzed using STRUCTURE software. Based on maximum-likelihood and *K* (Δ*K*)-values, the optimal number of groups was two (*K* = 2). As shown in Figure 4, the *O. longilobus* species could be separated from the *O. taihangensis* species, although some individuals were mixed between populations. When *K* = 3 (the second-best Δ*K-*value), 7 populations of *O. longilobus* still gathered into one group, while 12 populations of *O. taihangensis* were divided into two subgroups. Specifically, the WWS, YTS, WML, BJY, LSZT, and XYG populations were gathered together, whereas the other 6 populations FXF, SNS, GS, QLX, JYS, and DSC were clustered into another subgroup (Figure 4).

**FIGURE 5:**
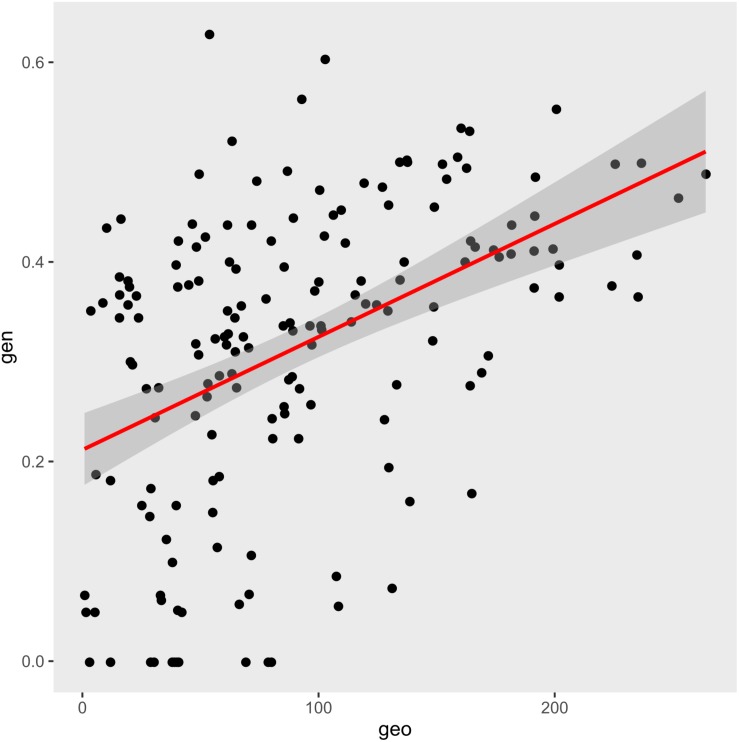
Geographic distance was positively correlated with genetic distance. The generalized linear regression model (GLRM): *y* = 0.0011*x* + 0.2117, *R*^2^ = 0.2082. Mantel test: *r* = 0.4565, *P* = 0.001.

The Mantel test was employed to investigate the correlations between genetic distances and geographic distances (Figure 5). Positive correlations between the genetic and geographic distances were clearly found in the GLRM (*r* = 0.4565, *P* = 0.001; *y* = 0.0011*x* + 0.2117, *R*^2^ = 0.2082) (Figure 5), which indicated that the genetic differentiation of the populations was linearly correlated with geographic distances.

## Discussion

### The Development of EST-SSRs Markers Based on *Opisthopappus* Transcriptome Database

Due to the lack of sufficient genetic information and effective molecular marker systems, the phylogenetic relationship between *Opisthopappus* (Asteraceae) species remains unclear and unresolved to a certain extent. SSR markers are considered to be one of the most important marker systems for plant genetic studies, with high polymorphisms, high abundance, co-dominance, and genome-wide distribution (Thakur and Randhawa, 2018; Zhang et al., 2018). Compared with the genomic SSRs, EST-SSRs are effective and useful markers for non-model plants, which are easily transferred between related species owing to these regions being more evolutionarily conserved than non-coding sequences (Varshney et al., 2005; Ellis and Burke, 2007; Tahan et al., 2009; Bazzo et al., 2018; Park et al., 2019).

The application of EST-SSR markers for genetic diversity, phylogenetic analyses, the construction of genetic maps, and the marker-assisted selection of important traits has recently been reported for several plant species (Dutta et al., 2011; Wang et al., 2013; Zhang et al., 2014; Jo et al., 2015; Huang et al., 2016; Vatanparast et al., 2016; Taheri et al., 2019). At present, the transcriptome sequencing technology (RNA-Seq) is a rapid, reliable, and cost-effective tool for the characterization of gene content, and the identification of polymorphic markers in non-model plants (Taheri et al., 2019; Zhang et al., 2019). An increasing number of successful examples have supported the strategy of using transcriptome data to predict SSR molecular markers (Zalapa et al., 2012; Han et al., 2018; Taheri et al., 2019).

For our work, among 33,974 assembled unigene sequences, 2644 potential EST-SSRs were identified, which represented ∼7.78% of the transcriptomic sequences (Supplementary Table S2), and the distribution density was one SSR per 10.30 kb. The SSRs frequency and distribution density were higher than some species, such as *Argyranthemum broussonetii* (2.3%, 27 kb) (White et al., 2016) and *Zingiber officinale* (2.7%, 25.2 kb) (Awasthi et al., 2017), and lower than *Arachis hypogaea* (17.7%, 3.3 kb) (Wang et al., 2018), *Curcuma longa* (14.6%, 5.3 kb; 14.9%, 5.2 kb; 20.5%, 4.8 kb) (Annadurai et al., 2013), and *Curcuma alismatifolia* (12.5%, 6.6 kb) (Taheri et al., 2019). Differences in the frequency of SSRs in ESTs could be partially attributed to the different genetic basis of various plant species, the size of the unigene assembly dataset, SSR search criteria, sequence redundancy, as well as the mining tools utilized (Kumpatla and Mukhopadhyay, 2005; Chen et al., 2015; Wei et al., 2016; Liu et al., 2018; Taheri et al., 2019).

Based on the number of bases, five different repeat motifs from mono- to penta-nucleotide were identified, in which the most abundant SSR motifs were mononucleotide repeats (45.39%), followed by trinucleotide repeats (37.52%), and dinucleotide repeats (15.51%). In contrast, hexa (1.48%) and penta (0.11%) were rare (Supplementary Table S2). This result was similar to previous findings of di- and tri-nucleotide motifs, which were reported as the more frequent SSR motif types within the transcriptome sequences of many other plants (Zheng et al., 2013; Zhang et al., 2014; Awasthi et al., 2017; Park et al., 2019; Taheri et al., 2019). In many organisms, the extensive distribution of trinucleotide repeats in coding sequences is an indicator of the effects of selection and evolution. Because open-reading frames do not disturb insertions and deletions within translated regions, this signifies that these SSRs do not change the coding frame of the gene regions (Feng et al., 2012; Bazzo et al., 2018). When there were variations in SSR lengths of other repetitions, they would lead to frame shifts and induce negative mutations, which might restrict the development of other motif types (Barrero et al., 2011; Park et al., 2019; Taheri et al., 2019).

Among the mononucleotide repeats, A/T repeats (95.08%) were far more prevalent than G/C repeats (4.92%), which was consistent with most plants (Gao et al., 2013; Yue et al., 2016; Feng et al., 2017; Taheri et al., 2019). The most abundant dinucleotide repeat was the AC/GT motif (65.37%), followed by AG/CT (22.93%) and AT/TA (10.73%), which was similar to previous findings in other *Chrysanthemum* species *Chrysanthemum indicum* (Han et al., 2018). Nevertheless, the most frequent repeats of this kind of motif might vary between different species, such as in *Zanthoxylum bungeanum* and *Oryza sativa* (Asp et al., 2007; Feng et al., 2017). For trinucleotide repeat motifs, the most abundant motif was ACC/GGT (22.50%), followed by ATC/ATG (20.11%), AAG/CTT (13.15%), AAC/GTT (12.07%), and AGC/CTG (11.09%), similar to reports on *C. indicum* (Han et al., 2018).

Abundant trinucleotide repeats generally encoded for threonine, isoleucine, lysine, asparagine, and serine, respectively, and except for isoleucine, these were primarily small hydrophilic amino acids. It is possible that the codon repeats corresponding to hydrophilic amino acids more easily tolerated selection pressures, which might eliminate codon repeats that encoded hydrophobic and basic amino acids (Katti et al., 2001; Han et al., 2018; Taheri et al., 2019). Consequently, the high occurrence level of these motifs was significant, as amino acids generated by them were observed in proteins to a high degree (Bazzo et al., 2018; Taheri et al., 2019; Zhang et al., 2019). The *Opisthopappus* species typically grows within the cliff cracks and rock gaps of the Taihang Mountains, have good drought tolerance, and are leanness-resistant (Hu et al., 2008; Chai et al., 2018). Abundant repetitions of the acids from the trinucleotide motif might explain these characteristics.

The 63 designed EST-SSR primers were obtained and verified, which offered an informative and applicable approach for the evaluation of genetic relationships between *Opisthopappus* species. Of the 63 primer pairs, 11 (17.5%) of the pair primers produced clear bands. This rate was relatively lower than that of previously reported species (Feng et al., 2017; Bazzo et al., 2018; Taheri et al., 2019; Zhang et al., 2019). Since there were no specificity or assembly errors following careful checking, the other 52 pair primers failed to generate the expected amplification size or non-amplification PCR products, which may have been due to the presence of introns and indels.

Meanwhile, the EST-SSR markers developed from *Opisthopappus* transcriptome data exhibited good transferability across different *Opisthopappus* species, indicating that these EST-SSR markers were useful tools for further genetic diversity analysis for this genus. The transferable nature of the EST-SSR markers between related species extended their usefulness in plant genetic studies (Varshney et al., 2005; Mathithumilan et al., 2013; Jia et al., 2016; Wang et al., 2018; Park et al., 2019).

### Genetic Differentiation and Phylogenetic Relationships Between *Opisthopappus* Species

*Opisthopappus*, which is an endemic and endangered genus that is geographically distributed in the Taihang Mountains, includes two species, according to the FRPS, while these two species are merged into a single species in the *Flora of China*. Therefore, the exact phylogenetic status of the *Opisthopappus* species remains under debate. Although the systematic classification and phylogenic research on *Opisthopappus* has been reported previously (Gao et al., 2011; Wang and Yan, 2013; Jia and Wang, 2015), *O. longilobus* populations are not well separated from *O. taihangensis* populations.

A number of reports have been published regarding the interspecific relationships between *Opisthopappus* species based on molecular markers (Wang, 2013; Wang and Yan, 2013); however, such research has been limited due to the lack of specific genomic information on *Opisthopappus*. Compared with other EST-SSR markers of plants (Kudapa et al., 2012; Onda et al., 2015; Wei et al., 2016; Li et al., 2018), the developed *Opisthopappus* transcriptomic SSR markers showed a high level of genetic diversity (Table 2), which indicated that the identified microsatellite loci possessed high allele numbers and heterozygosity, which provided efficient genetic markers for *Opisthopappus* populations.

Genetic diversity and fitness play a critical role in influencing the genetic structures of populations. High genetic diversity within populations is a successful strategy for adapting to various habitats and environmental conditions (Booy et al., 2000), which also increases the capacity of individuals plants, or populations thereof, to reach and colonize new habitats (Crawford and Whitney, 2010; Drummond and Vellend, 2012). Therefore, as one of the three forms of biodiversity, genetic diversity is recognized by the World Conservation Union (IUCN) as deserving conservation along with species and ecosystems (Reed and Frankham, 2003).

As an endangered perennial herb that is native to the Taihang Mountains, *Opisthopappus* genus populations are facing the prospect of completely disappearing in China. However, our results revealed that the genetic diversity of the populations evaluated with EST-SSR markers was more robust (Table 1). Moreover, *O. longilobus* populations exhibited a relatively higher level of genetic diversity than that of *O. taihangensis* populations (Table 1). *O. longilobus* primarily distributed along the northern regions of the Taihang Mountains, which has relatively low temperatures and high precipitation (Hu et al., 2008; Gong, 2010). In contrast, *O. taihangensis* is mainly distributed in the Southern Taihang Mountains (Hu et al., 2008; Gong, 2010). In comparison, the environmental conditions in which *O. longilobus* grown should be relatively harsher than that for *O. taihangensis*. Thus, the relatively inhospitable surroundings of *O. longilobus* might have cumulatively imbued it with high levels of genetic diversity.

In addition, based on the newly developed EST-SSR markers, it was found that there was a significant differentiation between *Opisthopappus* species (Figures 2–4). This differentiation might have resulted from long-term adaptions within a heterogeneous environment, as there are many gully valleys and gulches in the Taihang Mountains. Meanwhile, the complex topographies and diverse localized climates have also led to the varied surroundings of different species or populations.

The mantel test and GLRM (Figure 5) revealed a significant correlation between genetic and geographical distances; thus, geological impacts may have played a major role in driving and governing the genetic divergence of *Opisthopappus*. As new mountains formed, a long established population might have been broken up into spatially isolated subpopulations, which gradually accumulated genetic differences via adaptations to the local environment caused by this geographic segmentation (Meng et al., 2015). Originally, with changing monsoon patterns in East Asia during the Middle Miocene, the genus *Opisthopappus* have begun to differentiate into two major lineages: *O. longilobus* and *O. taihangensis* (Ye et al., 2019) and might have been homogeneously distributed across Northern China. Subsequently, with the uplift of Taihang Mountains and the glacial–interglacial cycles during Quaternary period (Gong, 2010; Zhang and Li, 2014), the population was spatially and geologically decollated into several different subpopulations situated in specific and distinct ecological niches, which initiated genetic differentiation and variation.

Furthermore, the Northern Taihang Mountains belong to a temperate continental semi-humid monsoon climate, while the Southern Taihang Mountains have a warm temperate continental monsoon climate (Wu et al., 1999; Gong, 2010). In the southern regions, the annual mean temperature is 12.7°C and the annual mean precipitation is 606.4 mm, whereas in the northern domains, the annual mean temperature is ∼10°C and the annual mean precipitation is 700 mm. Climatic and environmental diversity would result in geographical and ecological isolation between the species/populations, and promote the differentiation and speciation of *O. longilobus* and *O. taihangensis*. Therefore, with this accumulation of genetic differences, these isolated subpopulations began to evolve into genetically distinct lineages as the result of their adaptations to localized environmental conditions, to form the speciation pattern of *Opisthopappus*.

Clustering analysis (Figure 2) showed that all *Opisthopappus* populations were grouped into two major clusters, where *O. longilobus* populations gathered into one cluster, and *O. taihangensis* congregated into another (Figure 2). PCoA results also showed an obvious difference in the two groups (Figure 3), which was consistent with the clustering analysis (Figure 2). Moreover, BCA suggested that the best grouping number (*K*) was 2, and the second-best *K* was 3 based on the △*K* (Figure 4). All studied *O. longilobus* populations were gathered into one group in both *K* = 2 and *K* = 3. Nevertheless, the populations of *O. taihangensis* were inferred into two subgroups when *K* = 3. Therefore, we inferred that the *O. taihangensis* may retain a divergence potentiality to differentiation into distinct groups. The two inferences (*K* = 2 or *K* = 3) were similar and consistently indicated that the populations should be divided into two major groups, namely *O. taihangensis* and *O. longilobus*.

## Conclusion

For the current study, we provided a number of EST-SSR markers to elucidate the genetic relationships of *Opisthopappus* species, based on a *de novo* RNA-Seq assembly. To the best of our knowledge, this is the first attempt to develop SSR markers for this endemic and endangered genus. In the light of the above and previous results, the strategy for obtaining EST-SSRs and SNPs from the transcriptome proved to be efficient. Using the designed EST-SSRs and SNPs markers, a significant genetic differentiation was revealed between *O. longilobus* and *O. taihangensis* (Chai et al., 2018).

All of the studied *Opisthopappus* populations were separated into two distinct groups, which corresponded to *O. longilobus* and *O. taihangensis* species. These results indicated that *O. longilobus* and *O. taihangensis* should be regarded as two independent species, which was well supported by the FRPS. The developed and identified EST-SSR markers were shown to be efficient and significantly contributed to the genetic and evolutionary study of *Opisthopappus*.

## Data Availability Statement

The datasets generated for this study can be found in the NCBI and the GenBank accession numbers were SUB4002238 and PRJNA471103.

## Author Contributions

MC, HY, and ZW performed the experiments. HY, ZW, JW, and YG collected and analyed the transcriptome data. WH, EZ, WR, and HZ analyed the population genetics of Opishopappus species. MC, HY, and YW wrote the manuscript. MC, HY, YZ, GS, and YW edited the manuscript. All authors contributed to manuscript revision, read and approved the submitted version.

## Conflict of Interest

The authors declare that the research was conducted in the absence of any commercial or financial relationships that could be construed as a potential conflict of interest.

## Footnotes

1 Chinese version: http://www.iplant.cn/frps2019/frps?id=%e5%a4%aa%e8%a1%8c%e8%8f%8a%e5%b1%9e

2 English version: http://www.efloras.org/florataxon.aspx?flora_id=2&taxon_id=200163

3 http://www.upov.org/edocs/mdocs/upov/en/c/41/bmt_guidelines_proj_9.pdf
