# Supplementary material for: Genetic Divergence and Relationship Among Opisthopappus Species Identified by Development of EST-SSR Markers
Source: Front Genet. 2020 Feb 28;11:177. doi: 10.3389/fgene.2020.00177 (PMC7065708; doi:10.3389/fgene.2020.00177)
Supplement: Supplementary file 1 [file Table_1.DOCX]

| Table S1 Annotation results statistics of Unigenes | | |
| --- | --- | --- |
| Annotation in Database | Unigene No. | Percentage (%) |
| NR | 33974 | 100 |
| GO | 33974 | 100 |
| KO | 4857 | 14.3 |
| eggNOG | 10357 | 30.48 |
| Swiss-Prot | 30789 | 90.62 |
| In all database | 990 | 2.91 |
