# Supplementary material for: Genetic Divergence and Relationship Among Opisthopappus Species Identified by Development of EST-SSR Markers
Source: Front Genet. 2020 Feb 28;11:177. doi: 10.3389/fgene.2020.00177 (PMC7065708; doi:10.3389/fgene.2020.00177)
Supplement: Supplementary file 2 [file Table_2.DOCX]

Table S2 Summary of the EST-SSRs data of *Opisthopappus*

| Item | Number |
| --- | --- |
| Total number of sequences examined | 33974 |
| Total size of examined sequences (bp) | 27229229 |
| Total number of identified SSRs | 2644 |
| Number of SSR containing sequences | 2341 |
| Number of sequences containing more than 1 SSR | 272 |
| Number of SSRs present in compound formation | 143 |
| Mono-nucleotide | 1200 (45.39%) |
| Di-nucleotide | 410 (15.51%) |
| Tri-nucleotide | 992 (37.52%) |
| Tetra-nucleotide | 39 (1.48%) |
| Penta-nucleotide | 3 (0.11%) |
