# Supplementary material for: Genetic Divergence and Relationship Among Opisthopappus Species Identified by Development of EST-SSR Markers
Source: Front Genet. 2020 Feb 28;11:177. doi: 10.3389/fgene.2020.00177 (PMC7065708; doi:10.3389/fgene.2020.00177)
Supplement: Supplementary file 3 [file Table_3.DOCX]

Table S3 Independent-Samples T test of the genetic diversity parameters

|  | | Levene's test for  equality of variances | | T test for equality of means | | | | | | |
| --- | --- | --- | --- | --- | --- | --- | --- | --- | --- | --- |
|  |  | F | Sig. | t | df | Sig. (2-tailed) | Mean Difference | Std. Error Difference | 95% Confidence Interval of the Difference | |
|  |  |  |  |  |  |  |  |  | Lower | Upper |
| Na | Equal variances assumed | 2.540 | 0.1294 | -2.722038674 | 17.00 | 0.0145 | -0.3079 | 0.1131 | -0.5466 | -0.0693 |
|  | Equal variances not assumed |  |  | -2.3348264 | 8.13 | 0.0473 | -0.3079 | 0.1319 | -0.6112 | -0.0046 |
| Ne | Equal variances assumed | 0.135 | 0.7183 | -4.511733022 | 17.00 | 0.0003 | -0.1333 | 0.0295 | -0.1956 | -0.0710 |
|  | Equal variances not assumed |  |  | -4.318669524 | 11.10 | 0.0012 | -0.1333 | 0.0309 | -0.2012 | -0.0654 |
| H | Equal variances assumed | 2.065 | 0.1689 | -1.436265135 | 17.00 | 0.1691 | -0.0338 | 0.0235 | -0.0834 | 0.0158 |
|  | Equal variances not assumed |  |  | -1.221011294 | 7.94 | 0.2571 | -0.0338 | 0.0277 | -0.0977 | 0.0301 |
| I | Equal variances assumed | 0.176 | 0.6801 | -4.297227852 | 17.00 | 0.0005 | -0.1087 | 0.0253 | -0.1621 | -0.0553 |
|  | Equal variances not assumed |  |  | -4.012272637 | 10.29 | 0.0023 | -0.1087 | 0.0271 | -0.1688 | -0.0486 |
| PPB | Equal variances assumed | 4.002 | 0.0617 | -2.688580493 | 17.00 | 0.0155 | -0.1582 | 0.0588 | -0.2823 | -0.0341 |
|  | Equal variances not assumed |  |  | -2.306680873 | 8.13 | 0.0494 | -0.1582 | 0.0686 | -0.3159 | -0.0005 |

*N*a: observed number of alleles; *N*e: effective number of alleles; *H*: Nei’s genetic diversity; *I*: Shannon ‘s information index; *PPB*: the percentage of polymorphic loci/ band; F: F test; sig: significance; t: t test.
