# Supplementary material for: Genetic Divergence and Relationship Among Opisthopappus Species Identified by Development of EST-SSR Markers
Source: Front Genet. 2020 Feb 28;11:177. doi: 10.3389/fgene.2020.00177 (PMC7065708; doi:10.3389/fgene.2020.00177)
Supplement: Supplementary file 4 [file Image_1.pdf]

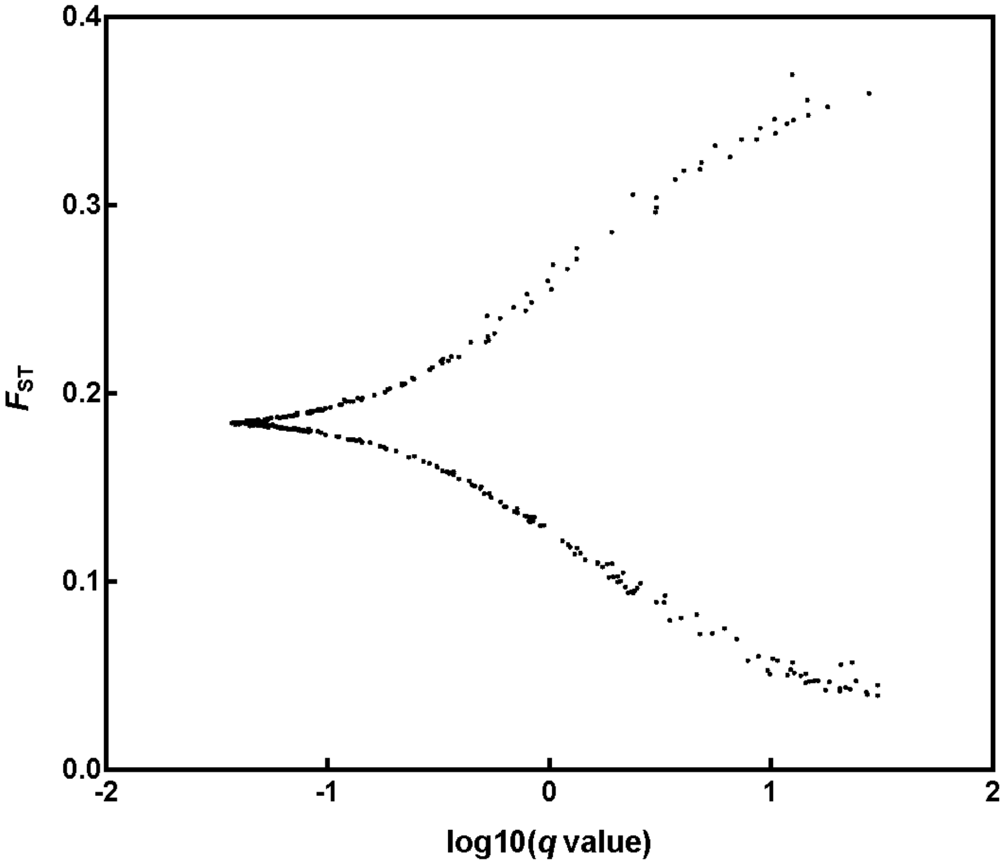

Figure S1 Neutrality tests for examining the F_{ST} distribution of microsatellite loci by the BayeScan approach.

The analysis shows that none of the remained alleles have extremely high (positive outlier) or low (negative outlier) F_{ST}, suggesting all alleles used in identifying species *O. longilobus* and *O. taihangensis* are neutral.
